# The characteristics and alteration of peripheral immune function in patients with multiple system atrophy

**DOI:** 10.3389/fneur.2023.1223076

**Published:** 2023-09-13

**Authors:** Minghui Wang, Zhaofen Yan, Jing Wang, Yujiao Yang, Qinqin Deng, Yixian Han, Liping Zhang, Huajun Yang, Jiali Pan, Mengyang Wang

**Affiliations:** Department of Neurology, Sanbo Brain Hospital, Capital Medical University, Beijing, China

**Keywords:** multiple system atrophy (MSA), immune function, CD19+ B lymphocyte, flow cytometry analysis, case-control study of cells

## Abstract

**Objective:**

Multiple system atrophy (MSA) is a degenerative disease. Immune dysfunction found to play a crucial role in the pathogenesis of this disease in the literature, while the characteristics of peripheral immune function remain unclear. This study aimed to investigate the characteristics and alterations of peripheral immune function in patients with MSA.

**Methods:**

A case–control study was conducted between January 2021 to December 2022 at SanBo Brain Hospital, Capital Medical University, Beijing, China. A total of 74 participants were recruited, including 47 MSA patients and 27 non-MSA participants. Peripheral blood samples were collected from each participant. A total of 29 types of immune cells were measured using the flow cytometry analysis technology. Single-factor analysis and multiple-factor analysis (multiple linear regression models) were performed to determine the differences and risk factors in immune cells between the MSA and non-MSA groups.

**Results:**

Alterations of the count or percentage of CD19+ B lymphocytes and CD3−CD56+ B lymphocytes in MSA patients were found in this study. The reductions of the count and percentage of CD19+ B lymphocytes were still robust after adjusting for variables of age, gender, body mass index, albumin, and hemoglobin. Furthermore, the reductions in the count and percentage of CD19+ B lymphocytes in the MSA patients were more significant in women and individuals aged 60 years old or above than in the non-MSA participants.

**Conclusion:**

Our findings suggested that MSA patients may be influenced by B lymphocytes, particularly CD19+ cells. Therefore, the reductions in immune cells should be considered in the diagnosis and treatment of MSA. Further studies are warranted to confirm and expand upon these findings.

## Graphical Abstract

MSA, multiple system atrophy. 
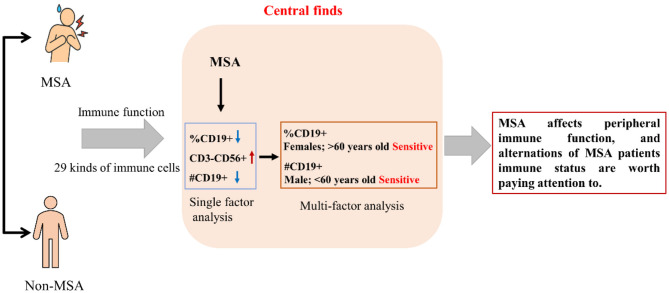

## 1. Introduction

Multiple system atrophy (MSA) is a rare and devastating neurodegenerative disorder, affecting both the autonomic and motor system (1), which is accompanied by symptoms of parkinsonism, cerebellar ataxia symptoms, pyramidal tract diseases, and other manifestations, first named in 1969 by Graham and Oppenheimer (2). The global epidemiological characteristics of MSA have been extensively studied in recent years. According to a meta-analysis of 58 studies published between 1996 and 2017, the prevalence of MSA ranged from 0.1 to 4.4 per 100,000 individuals worldwide, with the highest prevalence reported in Japan and Europe, and the lowest prevalence in Africa and South America (3, 4). Additionally, the incidence of MSA was found to be higher in men than that in women and was found to increase with age. The median age of onset for MSA was reported to be ~60 years old, while the median survival time after diagnosis was ~6 years (5). Whether the early diagnosis of MSA contributes to a complete cure is unclear, while a combination of simple measures and medications can help alleviate symptoms. However, the early diagnosis of MSA remains challenging due to the heterogeneity of its clinical presentation and the lack of specific biomarkers. Therefore, further research is needed to better understand the etiology and pathophysiology of MSA and to develop effective diagnostic and therapeutic strategies.

Although the exact etiology of MSA is still unclear, increasing evidence suggested that immune dysfunction probably plays a crucial role in the pathogenesis of this disease. In recent years, alterations of immune function in MSA have been reported, including the activation of glial cells, the dysregulation of cytokine levels, and the abnormal aggregation of alpha-synuclein protein (6). T lymphocytes are all involved in the pathogenesis (7), which can cross the blood–brain barrier to secrete lymphokine involved in MSA (8, 9). Furthermore, the detailed panoramic immune function changes in patients with MSA are not yet fully understood, particularly peripheric immune cells. Therefore, more global or systemic approaches are needed, which is necessary to reveal the immune function changes in MSA patients, and they further can provide a basis for identifying the etiology and developing potential therapeutic targets. In addition, there were several shared genetics (e.g., C7 gene) of MSA and inflammatory bowel disease (10), which indicated that the study of MSA immune function alteration probably also has implications for inflammatory bowel disease. This case–control study enrolled 74 participants to reveal the peripheric immune function alteration in patients with MSA and related influencing factors, which would provide a better understanding of the immune pathogenesis of MSA and a basis for exploring treatment targets and influencing factors.

## 2. Materials and methods

### 2.1. Study design and population

This case–control study enrolled patients diagnosed with MAS according to the diagnostic criteria of MSA (11) between January 2021 to December 2022 at SanBo Brain Hospital, Capital Medical University, Beijing, China, which is a hospital specializing in the treatment of neurological diseases. The participants of the non-MSA group were selected from individuals who were without health problems. The participants were screened based on the following criteria: (a) 18 years old or above; (b) not with autoimmune, inflammatory conditions, recent vaccination, immunomodulatory medication, or taking medicine affecting immune function; (c) diagnosed with MSA (MSA group) or non-MSA (non-MSA group) based on the diagnostic criteria; and (d) no history of using anti-inflammatory drugs 3 months before enrollment. The MSA patients were diagnosed based on the diagnostic criteria (11), which was widely applied globally. Briefly, the diagnostic criteria for MSA include the presence of autonomic dysfunction, symptoms of parkinsonism, and cerebellar ataxia, with a combination of supportive features, such as rapid disease progression, poor response to levodopa, and the presence of specific neuropathological findings. The information on the equivalent daily dose of levodopa (LEDD) dose was also collected for MSA patients. The participants in the non-MSA group were disease free and were the individuals who went to the hospital for routine health examinations. The demographic information [sex, age, and body mass index (BMI)] was extracted from the medical record management system. The variable of age was divided into ≤ 60 years old and >60 years old. The research project was approved by the Medical Ethics Committee of the National Center for SanBo Brain Hospital, Capital Medical University. All participants included in this study were written informed prior to enrollment consent. The Declaration of Helsinki and its amendments were followed in this study.

### 2.2. Indicators detection

Upon enrollment, venipuncture blood samples were collected from each participant by trained nurses. The albumin and hemoglobin were detected by laboratory professionals from Sanbo Brain Hospital, Capital University. The albumin category can be divided into normal (35–50 g/L) and abnormal (<35 or >50 g/L), while the hemoglobin category can also be divided into normal (male: 120–160 g/L, female: 110–150 g/L) and abnormal (male: <120 g/L, female: <110 g/L). Flow cytometry analysis (Beckman Coulter Flow Cytometer, USA) was used to measure the 29 peripheral immune cells within 4 h. The antibodies were purchased from BioLegend and Invitrogen. Briefly, the measurement method for immune function indicators included several steps, such as dye, antibody reaction, incubation, and cleanout. The procedure of each experiment was confirmed according to the instructions of each manufacturer. Cells were analyzed by the Beckman Count Flow Cytometer. The test for the 29 kinds of immune cells, including leukocyte (WBC), neutrophilic granulocyte percentage (%NEUT), lymphocyte percentage (%LYMPH), mononuclear leucocyte percentage (%MONO), neutrophil count (#NEUT), lymphocyte count (#LYMPH), monocyte count (#MONO), percentage of total T lymphocytes (CD3), percentage of total B lymphocytes (%CD19+), NK cell percentage (CD3−CD56+), percentage of NK-T cells (CD3+CD56+), percentage of helper T cells (CD3+CD4+), percentage of killer T cells (CD3+CD8+), percentage of helper/killer T cells (CD3+CD4+/CD3+CD8+), percentage of CD4+ initial T cells (CD4+CD45RA+), CD4+ effect and memory T cell percentage (CD4+CD45RO), CD8+ effect and memory T cell percentage (CD8+CD45RA+), CD8+ effect and memory T cell percentage (CD8+CD45RO), programmed death receptor 1 percent (PD1+), regulatory CD4+T cell percentage (CD4+CD25+CD127−), total T lymphocyte count (CD3+), total B lymphocyte count (#CD19+), NK cell count (CD3−CD56+), helper T cell count (CD3+CD4+), killer T cell count (CD3+CD8), CD4+ initial T cell count (CD4+CD45RA+), CD4+ effect and memory T cell count (CD4+CD45RO+), CD8+ initial T cell count (CD8+CD45RA+), and CD8+ effect and memory T cell count (CD8+CD45RO+), was conducted by Beijing Aokanghua Medical Laboratory Company.

### 2.3. Statistical analysis

The demographic characteristics and immune function variables were entered into Epidata 3.1 software. Double entry and logical checks were performed to ensure data accuracy. Statistical analyses were performed using SPSS 24.0 software for Windows 10. The continuous variables were tested for normality using the Shapiro–Wilk test. Mean ± standard deviation (*SD*) was used to express normally distributed data, while median (*M*) (*P*_25_, *P*_75_) was used to express skewed data. The categorical variables were expressed as *n* (%). Student's *t*-test and Wilcoxon rank-sum test were performed to compare the mean and median for continuous variables between the MSA and non-MSA groups, respectively, while the chi-square (χ^2^) test (including Fisher's exact probability method) was used in the categorical variables. The multiple linear regression model was performed to determine the difference in all 29 indicators for immune function between the MSA and non-MSA groups with age (≤ 60 years old and >60 years old), gender (male and female), BMI, albumin, and hemoglobin adjusted. Furthermore, to examine whether the results were robust enough to change the parameters, the subgroup analysis was performed to find high-risk groups. All statistical analyses were two-tailed, and statistical significance was defined as a *p*-value of <0.05.

## 3. Results

### 3.1. Characteristics of participants

A total of 74 participants were included in this study, while the characteristics for demographic, albumin, and hemoglobin are shown in Table 1, including 39 (52.7%) males and 35 (47.3%) females. The age of the participants ranged from 30 to 84 years old. The average age of participants in MSA and non-MSA was (58.83 ± 7.97) and (53.07 ± 11.47) years old, respectively. The majority of participants were <60 years old (64.9%). Among all the 74 participants enrolled in this study, 47 (63.5%) participants were at MSA status (MSA group), including 35 MSA-C and nine MSA-P, while 27 (36.5%) were at non-MSA status (non-MSA group). The average disease duration of MSA was (3.64 ± 1.92) years. A majority of MSA patients [41 (87.2%)] did not take LEDD, while the rest of the patients took LEDD at an average dose of 300 mg/day. Among all the participants, 26 males (66.7%) and 21 females (60.0%) were at MSA status, and the proportion of sex between the MSA and non-MSA groups was without significant difference (*p* > 0.05). The weighted proportions of MSA in <60 years and >60 years old were 43.8 and 23.1%, respectively (*p* > 0.05).

**Table 1:** Demographic characteristics across MSA status.

	***N* (%)**	**MSA group (%)**	**Non-MSA group (%)**	***t*/*χ^2^***	***p*-value^a^**
All participants	74 (100.0)	47 (63.5)	27 (36.5)	—	—
**Sex**				0.354	0.552
Male	39 (52.7)	26 (66.7)	13 (33.3)		
Female	35 (47.3)	21 (60.0)	14 (40.0)		
**Age**				3.110	0.078
≤ 60 years old	48 (64.9)	27 (56.3)	21 (43.8)		
>60 years old	26 (35.1)	20 (76.9)	6 (23.1)		
BMI (kg/m^2^)	—	25.11 ± 3.43	25.62 ± 2.35	—	0.479
Disease duration	—	3.64 ± 1.92	—	—	—
**Albumin (g/L)**				—	0.333^b^
Normal	4 (5.4)	45 (95.7)	25 (92.6)		
Abnormal	70 (94.6)	2 (4.3)	2 (7.4)		
**Hemoglobin (g/L)**				—	0.530^b^
Normal	72 (97.3)	45 (95.7)	27 (100.0)		
Abnormal	2 (2.7)	2 (4.3)	0 (0)		

^a^Calculated by the chi-square test.

^b^Fisher's exact probability method.

### 3.2. Immune function of the MSA and non-MSA groups

Overall, 29 kinds of peripheric immune cells were detected among the 74 participants, which are shown in Table 2. No significant differences were observed in the most (26 kinds) of cells, including WBC, %NEUT, %LYMPH, %MONO, #NEUT, #LYMPH, #MONO, CD3, CD3+CD56+, CD3+CD4+, CD3+CD8+, CD3+CD4+/CD3+CD8+, CD4+CD45RA+, CD4+CD45RO, CD4+CD45RA, CD8+CD45RO, PD1+, CD4+CD25+CD127−, CD3+, CD3−CD56+, CD3+CD4+, CD3+CD8, CD4+CD45RA+, CD4+CD45RO+, CD8+CD45RA+, and CD9+CD45RO+, while significant differences were observed in %CD19+, CD3−CD56+, and #CD19+ peripheric immune cells between the MSA and non-MSA groups (*p* < 0.05). The mean ±*SD* of CD19+ of participants was (8.05 ± 3.62) and (11.44 ± 3.87) in the MSA and non-MSA groups, respectively. The MSA group had a higher proportion (16.56 ± 9.30) of CD3−CD56+ than that of the non-MSA group (12.28 ± 7.59). The count of CD19+ in the MSA group (147.13 ± 76.77) count/μl was lower than that in the non-MSA group (228.94 ± 98.06) count/μl. Furthermore, the immune function was classified into categorical variables (normal and abnormal values). The abnormal rate of 29 indicators was compared between the MSA and non-MSA groups, which showed no significant differences in all the immune cells (*p* > 0.05; Supplementary Table S1). These results indicated that MSA status could be associated with the levels of immune cells although the alteration was light.

**Table 2:** Peripheric immune evaluation of the MSA and non-MSA groups by a numerical variable.

**Indicators**	**All participants**	**MSA group**	**Non-MSA group**	***p*-value^a^**
White blood cells (10^9^ cells/L)	6.26 ± 1.45	6.26 ± 1.38	6.26 ± 1.59	0.991
%NEUT (%)	59.54 ± 8.34	60.33 ± 8.54	58.15 ± 7.94	0.280
%LYMPH (%)	30.74 ± 7.64	29.84 ± 8.03	32.31 ± 6.79	0.184
%MONO (%)	7.09 ± 2.11	7.42 ± 2.26	6.50 ± 1.72	0.070
#NEUT (10^9^/L)	3.79 ± 1.27	3.84 ± 1.29	3.70 ± 1.25	0.637
#LYMPH (10^9^/L)	1.88 ± 0.47	1.82 ± 0.47	1.98 ± 0.47	0.148
#MONO (10^9^/L)	0.44 ± 0.16	0.46 ± 0.17	0.40 ± 0.15	0.143
CD3 (%)	59.31 ± 10.68	59.30 ± 10.66	59.33 ± 10.66	0.992
%CD19+ (%)	9.29 ± 4.04	8.05 ± 3.62	11.44 ± 3.87	<0.001^*^
CD3−CD56+ (%)	15.00 ± 8.90	16.56 ± 9.30	12.28 ± 7.59	0.046^*^
CD3+CD56+ (%)	3.45 (2.00, 7.10)	3.30 (1.60, 7.10)	3.90 (2.20, 9.90)	0.271^b^
CD3+CD4+ (%)	35.37 ± 9.33	36.49 ± 9.34	33.40 ± 9.15	0.172
CD3+CD8+ (%)	18.42 ± 9.09	17.80 ± 9.42	19.52 ± 8.56	0.437
CD3+CD4+/CD3+CD8+ (%)	2.60 ± 1.93	2.69 ± 1.57	2.45 ± 2.46	0.624
CD4+CD45RA+ (%)	11.57 ± 6.48	12.36 ± 6.97	10.10 ± 5.27	0.162
CD4+CD45RO (%)	1.42 ± 0.52	24.98 ± 6.90	23.70 ± 6.65	0.443
CD4+CD45RA (%)	14.47 ± 6.99	14.28 ± 7.69	14.82 ± 5.54	0.756
CD8+CD45RO (%)	12.85 ± 6.40	12.72 ± 7.12	13.08 ± 4.97	0.818
PD1+ (%)	8.60 ± 4.34	8.27 ± 4.47	9.18 ± 4.12	0.390
CD4+CD25+CD127− (%)	5.41 ± 2.13	5.28 ± 2.09	5.66 ± 2.22	0.465
CD3+ (count/μl)	1119.27 ± 369.16	1079.00 ± 361.68	1189.38 ± 378.37	0.218
#CD19+ (count/μl)	176.98 ± 93.33	147.13 ± 76.77	228.94 ± 98.06	<0.001^*^
CD3−CD56+ (count/μl)	276.25 ± 169.47	294.50 ± 172.00	244.49 ± 163.23	0.224
CD3+CD4+ (count/μl)	670.19 ± 262.55	670.79 ± 270.13	669.15 ± 253.85	0.980
CD3+CD8 (count/μl)	345.85 ± 191.70	318.50 ± 176.49	393.46 ± 210.63	0.106
CD4+CD45RA+ (count/μl)	220.27 ± 143.52	230.20 ± 152.22	201.61 ± 126.37	0.425
CD4+CD45RO+ (count/μl)	462.22 ± 174.23	455.57 ± 175.19	474.24 ± 175.28	0.664
CD8+CD45RA+ (count/μl)	264.64 ± 131.13	250.62 ± 127.70	291.00 ± 136.03	0.216
CD8+CD45RO+ (count/μl)	244.30 ± 149.47	235.40 ± 166.27	260.39 ± 114.25	0.498

^*^Significant differences compared to the non-MSA group (p <0.05).

^a^Calculated by the t-test.

^b^Calculated by the Mann–Whitney U-test.

As shown in Table 3, after adjusting for parameters of age, sex, BMI, albumin, and hemoglobin, multiple linear regression analysis was performed. All the variance inflation factors (*VIF*) were less than 10, which indicated that the correlation of the variables was acceptable. The results showed that MSA status was negatively associated with %CD19+ [β = −3.100, 95% *CI* (−5.150, −1.050)] and #CD19+ [β = −53.760, 95% *CI* (−98.520, −9.001)]. The %CD9+ and #CD9+ reduced by 30% (3.39%) and [81.81 (count/μl)] in the MSA group compared with those in the non-MSA group (Tables 2, 3). However, the correlations (*r*) of disease duration of MSA with %CD19+ and #CD19+ were 0.079 and 0.160 (both *p* > 0.05), respectively.

**Table 3:** Estimated of MSA group on immune evaluation compared to the non-MSA group.

**Indicators**	***B***	**β 95% *CI***	***p*-value**
WBC	0.196	−0.613, 1.005	0.631
%NEUT	−0.439	−1.007, 1.228	0.852
%LYMPH	0.594	−1.594, 1.782	0.778
%MONO	0.333	−0.789, 1.455	0.556
#NEUT	0.127	−0.599, 0.853	0.350
#LYMPH	0.070	−0.173, 0.313	0.566
#MONO	0.031	−0.053, 0.116	0.463
CD3	1.702	−2.394, 3.798	0.579
%CD19+	−3.100	−5.150, −1.050	0.004^*^
CD3−CD56+	2.596	−2.360, 7.552	0.300
CD3+CD56+	−0.320	−4.308, 3.667	0.873
CD3+CD4+	5.811	−0.822, 10.799	0.129
CD3+CD8+	−3.205	−8.252, 1.842	0.209
CD3+CD4+/CD3+ CD8+	0.606	−0.504, 1.716	0.280
CD4+CD45RA+	3.509	−0.340, 7.358	0.073
CD4+CD45RO	3.208	−0.594, 7.011	0.097
CD4+CD45RA	−2.713	−6.680, 1.255	0.177
CD8+CD45RO	−1.066	−4.883, 2.751	0.579
PD1+	−1.417	−3.909, 1.076	0.261
CD4+CD25+CD127−	−0.805	−2.033, 0.424	0.196
CD3+	53.177	−142.530, 248.884	0.589
#CD19+	−53.760	−98.520, −9.001	0.019^*^
CD3−CD56+	72.213	−23.384, 167.809	0.136
CD3+CD4+	125.589	−10.585, 261.764	0.070
CD3+CD8	−54.241	−161.645, 53.163	0.317
CD4+CD45RA+	79.427	−4.520, 163.374	0.063
CD4+CD45RO+	75.808	−15.529, 167.145	0.102
CD8+CD45RA+	−37.642	−114.528, 39.244	0.332
CD8+CD45RO+	−3.054	−91.892, 85.785	0.945

^*^Significant differences compared to the non-MSA group (p < 0.05).

Age and sex were adjusted.

### 3.3. Subgroup analysis

**Figure 1:**
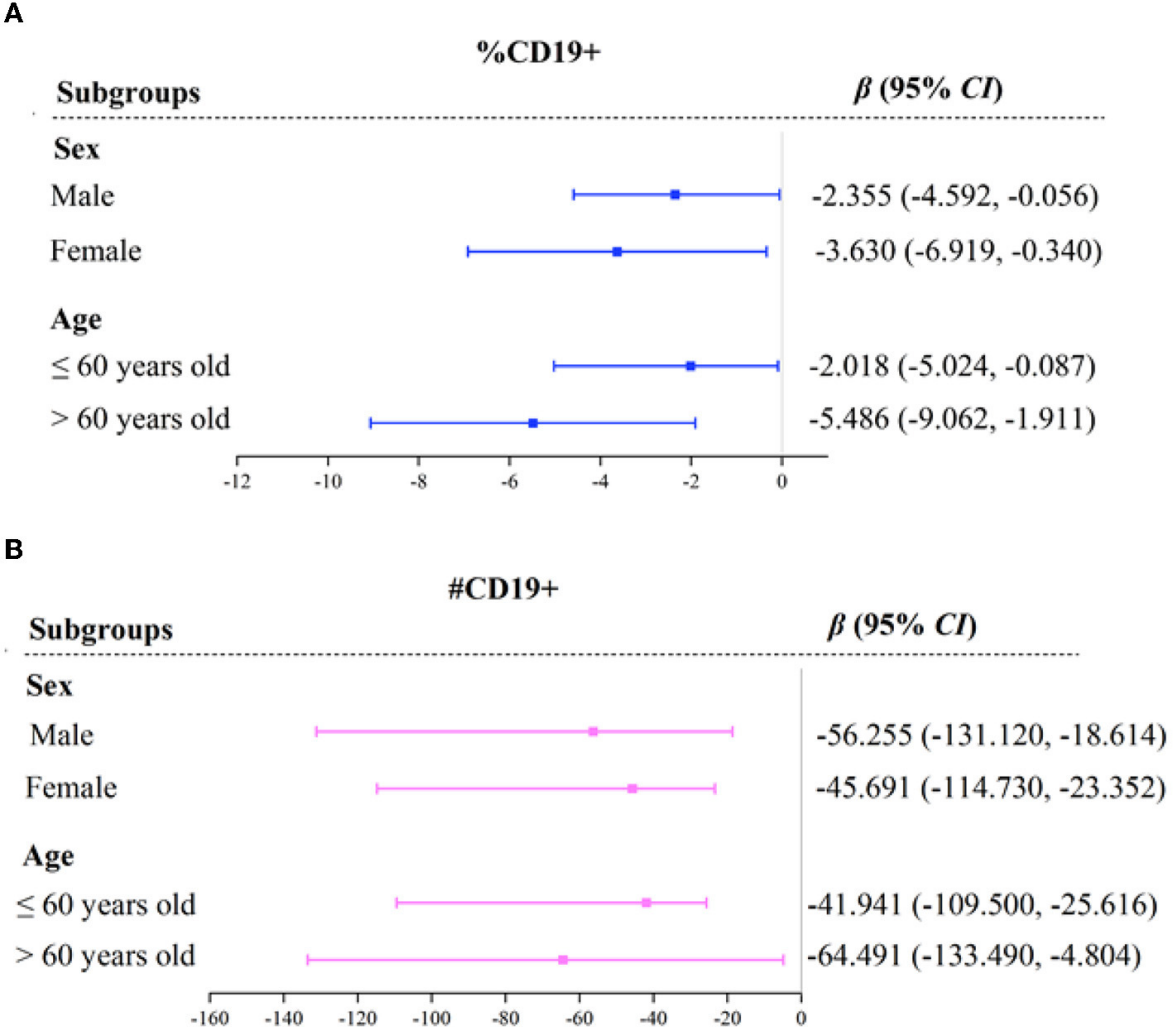
Forest plot of immune function indicators across MSA with subgroups. OR, odds ratio; CI, confidence interval. %CD19, percentage of total B lymphocytes **(A)**. #CD19, total B lymphocytes count **(B)**. Variables of sex, age, BMI, albumin, and hemoglobin, were adjusted in the model.

The subgroup analysis between immune function and MSA is shown in Figure 1 and Supplementary Table S2. Regardless of subgroups, MSA was negatively associated with immune function indicators of %CD19+ and # CD19+. The effects of MSA on %CD19+ were more significant in participants who were females [β = −3.630, 95% *CI* (−6.919, −0.340)] and older than 60 years old [β = −5.486, 95% *CI* (−9.062, −1.911)], while the effects on #CD19+ were more significant in participants who were males [β = −56.255, 95% *CI* (−131.124, −18.614)] and younger than 60 years old [β = −64.491, 95% *CI* (−133.487, −4.804)].

## 4. Discussion

The present study aimed to investigate the peripheric immune function alterations among patients with MSA and further aimed to explore the possible risk factors. The results showed that MSA patients had significantly different levels of peripheric immune function indicators compared to healthy non-MSA participants. In particular, the CD19+ B lymphocyte cells significantly decreased among patients with MSA, which was a major immune cell involved in the adaptive immune response.

The present study findings were consistent with previous studies that have suggested immune dysfunction may act a crucial role in the pathogenesis of MSA (12, 13). The levels of CD3+ and CD4+ T-lymphocytes and CD4+/CD8+ ratios among MSA patients were higher than those of healthy controls, which suggested that peripheral immune activation would probably be related to the prevalence of MSA in the Chinese patient population (14). The activation of glial cells, dysregulation of cytokine levels, and abnormal aggregation of alpha-synuclein protein have been reported to be involved in the immune pathogenesis of MSA (15, 16). The present study provides a more comprehensive understanding of the immune dysfunction in MSA by revealing alterations in the levels of various immune cells. The level of CD3−CD56+NK cells was significantly higher in the MSA patients than that in the non-MSA participants, which indicated that the MSA patients were in a pro-inflammatory status and the anti-inflammatory therapy might be beneficial to MSA (17).

The decreased levels of CD19+ B lymphocyte cells in MSA patients may reflect the impairment of the adaptive immune response in these patients. The adaptive immune response played a critical role in identifying and eliminating foreign pathogens, as well as in controlling the growth and spread of cancer cells (18, 19). The decreased levels of B lymphocyte cells in MSA patients may result in a weakened ability to mount an effective immune response against pathogens or cancer cells. This may contribute to the increased susceptibility of MSA patients to infections and cancer, which have been reported in previous studies (20). A higher neutrophil-to-lymphocyte ratio (NLR) increased the risk of mortality with MSA and predicted short survival in a cohort study, especially in the MSA with cerebellar patients and in men (21).

Recent studies have also implicated regulatory B cells (Bregs) in the pathogenesis of autoimmune diseases (22). Bregs were a subset of B cells that have immunosuppressive properties and were capable of regulating T cell response (23). In MSA patients, it was possible that the decrease in CD19+ B lymphocytes was due to a reduction in Bregs. In addition, the count of lymphocytes reflected the nutritional status predicting the prognosis of many diseases (24, 25), while the nutritional indicators did not have a significant difference between the two groups. Moreover, the autonomic nervous system was reported to influence the distribution of lymphocytes previously, while most patients with MSA exhibited autonomic dysfunction (26). The alterations in neuroregulatory factors in MSA patients probably affect the distribution of immune cells (27, 28). Future studies should investigate the role of Bregs in MSA and determine whether targeting these cells could be a potential therapeutic strategy. Another possible explanation for the decrease in CD19+ B lymphocytes in MSA patients is the involvement of alpha-synuclein (29), a protein that is found in high levels in the brains of MSA patients. Alpha-synuclein has been shown to have immunomodulatory effects and may play a role in regulating B cell populations. It is possible that the accumulation of alpha-synuclein in MSA patients leads to a reduction in CD19+ B lymphocytes. It has been reported that B lymphocytes were involved in the progress of Parkinson's disease (PD), a kind of neurodegenerative disease that shared a similar performance to MSA-P (30). B lymphocytes were also decreased in PD, which was protective in PD (31). While alpha-synuclein was possible to act a role to increase in PD early disease (30–32). Therefore, the role of B lymphocytes and alpha protruding protein in the pathogenesis of MSA probably needs to be focused in future.

The present study also investigated the possible influencing factors of immune dysfunction in MSA patients. The results of this study showed that age and sex were associated with the levels of immune function indicators in MSA patients. Specifically, older age and females were associated with lower counts of CD19+ B lymphocyte cells in MSA patients, while younger age and males were associated with a lower percentage of CD19+ B lymphocyte cells. This suggested that immune dysfunction in MSA may be a progressive process that worsens with age and sex (33, 34).

The present study also had some limitations that should be acknowledged. First, the sample size of this study was relatively small since MSA was a relatively rare disease, which may limit the generalizability of the findings. Second, this study only investigated the peripheric immune function alterations in MSA patients at a single time point, which may not fully reflect the dynamic changes in immune function over time. The subtypes of MSA were not distinguished in this study due to the lack of sufficient participants. Future longitudinal studies with larger sample sizes are needed to confirm the findings of this study and to investigate the dynamic alterations in immune function in MSA patients over time. Third, autoimmune skin diseases, such as bullous pemphigoid, were not found in patients, although studies reported that bullous pemphigoid (BP) was associated with MSA, at a latency of 4–6 years from the MSA onset (35).

In conclusion, the present study revealed alterations in the levels of various immune cells in MSA patients, indicating peripheric immune dysfunction in these patients. The decreased levels of CD19+ B lymphocyte cells probably reflect the impairment of the adaptive immune response.

## Data availability statement

The original contributions presented in the study are included in the article/Supplementary material, further inquiries can be directed to the corresponding author.

## Ethics statement

The studies involving humans were approved by Ethics Committee of Sanbo Brain Hospital, Capital Medical University. The studies were conducted in accordance with the local legislation and institutional requirements. The participants provided their written informed consent to participate in this study.

## Author contributions

MiW and MeW conceptualized and designed the study. MiW completed the statistical analyses, charting, and drafted the initial manuscript. YY, QD, HY, YH, LZ, and JP participated in the collection of patient information. ZY, JW, and MeW reviewed and revised the manuscript. All authors read and approved the final manuscript.
