# Supplementary material for: The characteristics and alteration of peripheral immune function in patients with multiple system atrophy
Source: Front Neurol. 2023 Sep 13;14:1223076. doi: 10.3389/fneur.2023.1223076 (PMC10525398; doi:10.3389/fneur.2023.1223076)
Supplement: Supplementary file 1 [file Table_1.DOCX]

**Table S1.** Immune evaluation of the MSA and control groups by abnormal rate.

| Indicators | All participants | MSA group (%) | Control group (%) | *p*-value ^a^ |
| --- | --- | --- | --- | --- |
| WBC | 5 (6.8) | 3 (6.4) | 2 (7.4) | 0.997 ^b^ |
| %NEUT | 15 (20.3) | 11 (23.4) | 4 (14.8) | 0.376 |
| %LYMPH | 13 (17.6) | 10 (21.3) | 3 (11.1) | 0.269 ^b^ |
| %MONO | 2 (2.7) | 2 (4.3) | 0 (0) | 0.530 ^b^ |
| #NEUT | 3 (4.1) | 2 (4.3) | 1 (3.7) | 1.000 ^b^ |
| #LYMPH | 0 (0) | 0 (0) | 0 (0) | — |
| #MONO | 0 (0) | 0 (0) | 0 (0) | — |
| CD3 | 13 (17.6) | 8 (17.0) | 5 (18.5) | 1.000 |
| %CD19+ | 14 (18.9) | 12 (25.5) | 2 (7.4) | 0.555 |
| CD3-CD56+ | 16 (21.6) | 7 (14.9) | 9 (33.3) | 0.064 |
| CD3+CD56+ | 48 (64.9) | 30 (63.8) | 18 (64.9) | 0.806 |
| CD3+CD4+ | 20 (27.0) | 12 (25.5) | 8 (29.6) | 0.702 |
| CD3+CD8+ | 32 (43.2) | 23 (48.9) | 9 (33.3) | 0.192 |
| CD3+CD4+/CD3+CD8+ | 29 (39.2) | 22 (46.8) | 7 (25.9) | 0.076 |
| CD4+CD45RA+ | 40 (54.1) | 26 (55.3) | 14 (51.9) | 0.773 |
| CD4+CD45RO | 33 (44.6) | 23 (48.9) | 10 (37.0) | 0.322 |
| CD4+CD45RA | 29 (42.6) | 20 (42.6) | 9 (33.3) | 0.434 |
| CD8+CD45RO | 25 (33.8) | 15 (31.9) | 10 (37.0) | 0.654 |
| PD1+ | 52 (70.3) | 36 (76.6) | 16 (59.3) | 0.116 |
| CD4+CD25+CD127- | 3 (4.1) | 1 (2.1) | 2 (7.4) | 0.550 ^b^ |
| CD3+ | 25 (33.8) | 19 (40.4) | 6 (22.2) | 0.111 |
| #CD19+ | 14 (18.9) | 12 (25.5) | 2 (7.4) | 0.055 ^b^ |
| CD3-CD56+ | 21 (28.4) | 10 (21.3) | 11 (40.7) | 0.074 |
| CD3+CD4+ | 21 (28.4) | 15 (31.9) | 6 (22.2) | 0.373 |
| CD3+CD8 | 26 (35.1) | 17 (36.2) | 9 (33.3) | 0.806 |
| CD4+CD45RA+ | 39 (52.7) | 25 (53.2) | 14 (51.9) | 0.912 |
| CD4+CD45RO+ | 26 (35.1) | 19 (40.4) | 7 (25.9) | 0.208 |
| CD8+CD45RA+ | 24 (32.4) | 17 (36.2) | 7 (25.9) | 0.365 |
| CD9+CD45RO+ | 29 (39.2) | 15 (31.9) | 14 (51.9) | 0.091 |

^a^ Calculated by chi-square test.

^b^ Calculated by Fisher’s exact probability method.
