# Supplementary material for: The characteristics and alteration of peripheral immune function in patients with multiple system atrophy
Source: Front Neurol. 2023 Sep 13;14:1223076. doi: 10.3389/fneur.2023.1223076 (PMC10525398; doi:10.3389/fneur.2023.1223076)
Supplement: Supplementary file 2 [file Table_2.DOCX]

**Table S2.** Estimated of MSA on immune evaluation compared to the control groups.

| Indicators | *OR* | *OR* 95% *CI* | *p*-value |
| --- | --- | --- | --- |
| WBC | 1.249 | 0.177, 8.834 | 0.824 |
| %NEUT | 0.545 | 0.151, 1.968 | 0.354 |
| %LYMPH | 0.473 | 0.114, 1.960 | 0.302 |
| %MONO | — | — | — |
| #NEUT | 0.734 | 0.060, 8.981 | 0.808 |
| #LYMPH | — | — | — |
| #MONO | — | — | — |
| CD3 | 1.593 | 0.389, 6.523 | 0.517 |
| %CD19+ | 0.225 | 0.044, 1.154 | 0.074 |
| CD3-CD56+ | 2.801 | 0.859, 9.130 | 0.088 |
| CD3+CD56+ | 1.224 | 0.440, 3.401 | 0.699 |
| CD3+CD4+ | 1.510 | 0.493, 4.620 | 0.470 |
| CD3+CD8+ | 0.533 | 0.195, 1.458 | 0.220 |
| CD3+CD4+/CD3+CD8+ | 0.423 | 0.147, 1.212 | 0.109 |
| CD4+CD45RA+ | 0.818 | 0.309, 2.168 | 0.686 |
| CD4+CD45RO | 0.522 | 0.186, 1.459 | 0.215 |
| CD4+CD45RA | 0.765 | 0.270, 2.172 | 0.615 |
| CD8+CD45RO | 1.301 | 0.469, 3.611 | 0.613 |
| PD1+ | 0.402 | 0.135, 1.199 | 0.102 |
| CD4+CD25+CD127- | 7.298 | 0.467, 11.877 | 0.154 |
| CD3+ | 0.479 | 0.155, 1.478 | 0.201 |
| #CD19+ | 0.253 | 0.046, 1.407 | 0.116 |
| CD3-CD56+ | 2.390 | 0.819, 6.975 | 0.111 |
| CD3+CD4+ | 0.731 | 0.234, 2.291 | 0.591 |
| CD3+CD8 | 0.991 | 0.354, 2.775 | 0.987 |
| CD4+CD45RA+ | 1.014 | 0.384, 2.680 | 0.978 |
| CD4+CD45RO+ | 0.596 | 0.202, 1.755 | 0.347 |
| CD8+CD45RA+ | 0.775 | 0.257, 2.335 | 0.651 |
| CD9+CD45RO+ | 2.559 | 0.929, 7.046 | 0.069 |
